# TGF-β gene polimorphisms as risk factors for asthma control among clinic patients

**DOI:** 10.1186/s12950-021-00294-4

**Published:** 2021-10-07

**Authors:** Panek Michał, Stawiski Konrad, Kuna Piotr

**Affiliations:** 1 Department of Internal Medicine, Asthma and Allergy, Medical University of Lodz, ul. Kopcińskiego 22, 90-153 Łódź, Poland; 2 Department of Biostatistics and Translational Medicine, Medical University of Lodz, Lodz, Poland

**Keywords:** Asthma, TGFβ1, TGFβ2 and TGFβ3 gene, SNPs, Inflammation, Asthma control, The asthma control test (ACT™)

## Abstract

**Background:**

TGF-β and its receptors play a crucial role in asthma pathogenesis, bronchial hyperreactivity, and bronchial remodeling. Expression of isoforms 1–3 of TGFβ cytokine is influenced by tagging polymorphisms in the TGFβ1, TGFβ2 and TGFβ3 gene, and these SNPs may be associated with the risk of asthma development and severity as well as with other diseases. Polymorphic forms of TGF-β1, TGF-β2 and TGF-β3 genes regulate the degree of bronchial inflammation, deterioration of lung functional parameters in spirometry and elevated level of total IgE. All this results in intensification of disease symptoms. According to current GINA 2020 guidelines, the Asthma Control Test (ACT™) should be applied to assess asthma symptoms.

**Methods:**

An analysis of polymorphisms localized in TGF-β1, TGF-β2 and TGF-β3 genes was conducted on 652 DNA samples with an application of the MassARRAY® system using the mass spectrometry technique MALDI TOF MS. The degree of asthma control was evaluated with ACT™.

**Results:**

The occurrence of the T / C genotype in rs8109627 (*p* = 0.0171) in the TGF-β1 gene is significantly associated with a higher ACT result (controlled asthma) in a multivariate linear regression analysis model after using backward stepwise selection of variables. In addition, in the linear model for prediction of ACT score we showed SNP rs8109627 (*p* = 0.0497) in the TGF-β1 gene (improvement of the disease control - controlled asthma) and rs2796822 (*p* = 0.0454) in the TGF-β2 gene (deterioration of the diseases control - uncontrolled asthma) significantly modify the degree of asthma control.

**Discussion:**

We described clinical significance of two SNPs in two genes TGF-β1 and TGF-β2, as yet unknown. We proved that the use of both genotypes and MAC allows to create a moderately correct prognostic model which is about 70% efficient on the entire set of analyzed SNPs in TGF-β1, TGF-β2, and TGF-β3 genes.

**Supplementary Information:**

The online version contains supplementary material available at 10.1186/s12950-021-00294-4.

## Background

Asthma is a chronic, inflammatory and heterogeneous respiratory disease. Many genes, inflammatory cells, and substances secreted by them are involved in the etiopathogenesis of this disease. The clinical picture depends on complex gene-gene and gene-environment interactions. These components make asthma a dynamic disease, and its classifications can change both during treatment and irrespective of it. The etiology of bronchial asthma is multifactorial. The disease develops under the influence of variables, which, according to the report of the *Global Strategy for Asthma* Management and *Prevention* (GINA) can be divided into those which induce asthma and those which trigger its clinical symptoms. According to current GINA 2019 guidelines, the Asthma Control Questionnaire (ACQ) or the Asthma Control Test (ACT) can be used to assess its symptoms. The severity of the disease is assessed not based on the intensity of symptoms before the treatment is initiated, but only after several months of the treatment, when the level of intensity necessary to achieve and maintain asthma control has been established [1–4]. TGF-β (transforming growth factor β) is the most important gene responsible for bronchial hyperreactivity and the development of structural changes in bronchial walls is [5–8]. The key role of TGF-β as a growth factor responsible for prolonged reparatory processes and activation of myofibroblasts has been demonstrated in many studies on airway inflammation and its bronchial remodeling in asthma [5–12]. Increased TGFβ expression has been observed in asthma, chronic obstructive pulmonary disease, idiopathic pulmonary fibrosis, cystic fibrosis, systemic scleroderma, non-alcoholic steatohepatitis, renal failure, myocardial infarction, or heart failure. It should be mentioned here that deregulation of TGFβ cell signaling homeostasis is the main mechanism initiating and regulating fibrosis progression in various tissues. TGFβ is a pleiotropic cytokine that regulates cell proliferation, contributes to epithelial-to-mesenchymal transition (EMT), suppresses the function of immune cells, compromising immune response, contributes to the conversion of fibroblasts to myofibroblasts, and causes overproduction of extracellular matrix (ECM) in tissues undergoing fibrosis. TGFβ upregulates the expression and synthesis of many matrix proteins, primarily through the recruitment of myofibroblasts. Proteins upregulated by TGFβ include collagens I - V, basement membrane proteins (laminin, entactin, perlecan) and ECM proteins (fibronectin, osteopontin, thrombospondin, tenascin, osteonectin/SPARC, elastin, biglycan, decorin, and hyaluronan). Additionally, at the early stages of fibrosis, TGFβ stimulates myofibroblasts and other stromal cells to enhance the synthesis of collagen crosslinking enzymes, particularly lysyl oxidase, which increases the rigidity of the collagen network. Moreover, TGFβ downregulates the synthesis of matrix-depleting proteins, such as matrix metalloproteinases (MMP-1, − 8, − 13). As a result, the increase in matrix protein synthesis and decrease in matrix proteinase activity, owing to the TGF β activity, contribute to the remodeling of the bronchial ECM and can result in a fibrotic response [11–16]. Polymorphic forms of the TGFβ gene can significantly contribute to the development of asthma, induce disease progression and its complications, and have a significant clinical impact on symptom control. Single Nucleotide Polymorphism (SNP) rs8179181 in the TGFβ1 gene induces childhood asthma and atopy development. It increases the risk of a severe course of the disease and correlates with high levels of TGFβ1 mRNA [17]. rs4803455 has been reported to increase the risk of asthma development. This SNP down-regulates lung function and induces airway remodeling in asthma [18]. rs1800469 in the TGF-β1 gene promoter increases the plasma level of TGF-β1 and the level of total IgE. It may also reduce the risk of progression of bronchial remodeling and inhibit the development of asthma [12, 19–21]. rs11083616 correlates with bronchial obstruction and with airway wall phenotypes - airway wall thickness. The presented SNP is an important marker of the development of all obstructive diseases [22]. The role of rs8109627 in the TGFβ1 gene has not been explicitly and precisely described in the etiopathogenesis of asthma. SNPs tagging in the TGFβ2 gene (rs10495098, rs17047703, rs17558745, rs2799085, rs2009112, rs10482751, rs2027567, rs10779329, rs2796821, rs2796822, rs4846479, rs2798631, rs10863399) and the TGFβ3 gene (rs4903359, rs3917187, rs2284792, rs2268626) have not been analyzed as risk factors for development of obstructive diseases [23]. Table 1 presents detailed characteristics of TGFβ1, TGFβ2, and TGFβ3 genes and their SNPs.

**Table 1:** Presentation of SNPs TGFβ genes on the base of the NCBI gene database, available on https://www.ncbi.nlm.nih.gov

Name/Gene ID	Description	Location	Aliases	MIM	SNPs	Role in asthma
TGFB1 ID: 7040	transforming growth factor beta 1 [*Homo sapiens* (human)]	Chromosome 19, NC_000019.10 (41,330,323..41353922, complement)	CED, DPD1, IBDIMDE, LAP, TGF-beta1, TGFB, TGFbeta	190,180	rs8109627, rs8179181, rs4803455, rs1800469, rs11083616	dimerization and phosphorylation of receptor-activated Smad proteins
TGFB2 ID: 7042	transforming growth factor beta 2 [*Homo sapiens* (human)]	Chromosome 1, NC_000001.11 (218,345,284..218444619)	G-TSF, LDS4, TGF-beta2	190,220	rs10495098, rs17047703, rs17558745, rs2799085, rs2009112, rs10482751, rs2027567, rs10779329, rs2796821, rs2796822, rs4846479, rs2798631, rs10863399	expressed by eosinophils; predominant in severe allergic asthma
TGFB3 ID: 7043	transforming growth factor beta 3 [*Homo sapiens* (human)]	Chromosome 14, NC_000014.9 (75,958,061..75983011, complement)	ARVD, ARVD1, LDS5, RNHF, TGF-beta3	190,230	rs4903359, rs3917187, rs2284792, rs2268626	lung cells become TGF-β3 positive under inflammatory conditions

https://www.ncbi.nlm.nih.gov/gene/?term=TGFB1

https://www.ncbi.nlm.nih.gov/gene/?term=TGFB2

https://www.ncbi.nlm.nih.gov/gene/?term=TGFB3

### Aims

Our purpose was to determine whether genotypes of MAC/SNP TGFβ1, TGFβ2, and TGFβ3 are related to the level of asthma control, measured with the application of the Asthma Control Test (ACT™) in asthmatics and healthy controls.

## Methods

### Consent of the bioethics committee

The study was approved by the local ethics committee (Consent of Research Review Board at the Medical University of Lodz, Poland, No RNN/133/09/KE). At the commencement of the study, the participants were invited to get involved voluntarily. Before the enrolment, written informed consent was obtained from each patient.

### Subjects

Asthma diagnosis was established according to GINA (The Global Initiative For Asthma) recommendations, based on clinical asthma symptoms and a lung function test. The level of asthma severity and control was determined on the basis of the GINA Report Guidelines. All the participants underwent subjective examinations (structuralized anamnesis including, besides the element of subjective examination, also an analysis of factors such as: gender, obesity, tobacco smoking, duration of bronchial asthma, allergy to house dust mites, animal fur, mold spores, cockroaches allergens, hypersensitivity to non-steroid anti-inflammatory drugs (NSAIDs), etc. Results of pulmonary function tests and allergological tests were obtained from individual medical records of the patients. If results of spirometry or allergological tests were not available, such examinations were additionally performed during the recruitment visit. Subjects are suffering from clinically significant exacerbations and using drugs that might induce resistance to glucocorticoids (such as rifampicin, phenobarbital, phenytoin, ephedrine), subjects with signs of viral infections, either generalized or affecting the respiratory tract, as well as subjects failing to comply with the doctor’s recommendations, were excluded from the patient group. The control arm included a group of healthy adults who met the following criteria: no history or symptoms of either bronchial asthma or other pulmonary diseases, no history or symptoms of allergy, no history or symptoms of atopic dermatitis, no history or signs of hypersensitivity to aspirin, negative results of skin tests for 12 common allergens, no first-degree relatives with bronchial asthma or atopic disorders. Spirometry tests were conducted in the Outpatient Clinic according to ERS (European Respiratory Society)/ATS (American Thoracic Society) standards, and allergological tests according to EAACI (European Academy of Allergy and Clinical Immunology) guidelines [12, 19–21, 24–26].

Note: The authors of the article for this scientific project used their own patient database containing detailed statistical and genetic characteristics used in previous publications. However, this manuscript does not present results published earlier in any other study (the article contains original results not published elsewhere).

Six hundred fifty-two subjects were included in the study. Of this number, there were 345 asthmatic patients at the average age of 48.6 years and 307 healthy subjects at 46.0 years. Detailed patients’ characteristics were presented in Table 2.

**Table 2:** Clinical characteristics of the recruited cohort and spirometric characteristics of the studied groups

Characteristics of the studied groups	Asthmatic Patients (*n* = 345)	Healthy Controls (*n* = 307)	*p*-value
Age [years]	48.6 ± 15.4	46.0 ± 16.3	0.04
Sex	Females: 222 Males: 123	Females: 197 Males: 110	0.96
FEV1(%)	75.81 ± 21.37	95.83 ± 19.95	< 0.001
FVC(%)	93.52 ± 18.78	101.63 ± 17.69	< 0.001
FEV1/FVC(%)	83.25 ± 14.71	95.88 ± 10.05	< 0.001
Smoking pack years	5.82 ± 10.98	5.68 ± 11.71	0.88

FEV1 (forced expiratory volume in 1 s) expressed in %, FEV1% (A/N% - percentage ratio of the measured to expected value) expressed as a percent of the expected value; FVC (forced vital capacity) expressed in %, FVC% (A/N% - percentage ratio of the measured to expected value) expressed as a percent of the predicted value; FEV1% FVC index (FEV1 to FVC ratio - forced vital capacity) expressed in %

The whole group of patients participating in the study was stratified and subjected to statistical analysis. The genotyping was performed by two investigators who were unaware of the phenotypes.

### Genomic DNA extraction and SNPs analysis (MassARRAY® system)

Venous blood samples were collected from the participants onto EDTAK3, and DNA was obtained from peripheral blood leukocyte fraction. The genetic material was isolated using QIAamp DNA Blood Mini Kit (QIAGEN Inc.) according to guidelines provided by the manufacturer (12,13,17,18,19).

An analysis of polymorphisms localized in TGF-β1, TGF-β2 and TGF-β3 genes was conducted on 652 DNA samples with the application of the MassARRAY® system (Bionanopark, Lodz, Poland), using the mass spectrometry technique (MALDI TOF MS – matrix-assisted laser desorption ionization–time-of-flight mass spectrometry), dedicated to nucleic acids.

The authors measured the output level of genomic human DNA and diluted DNA to obtain final cDNA levels = 15 [ng/μl]. In samples with a DNA level lower than 15 [ng/μ], a sufficient undiluted amount of sample was used for analytical purposes. The absorbance ratio A260/A280, identified as a degree of DNA impurities, ranged from 1.7 to 2.0. The DNA levels were measured in all the studied samples.

The first stage of laboratory tests involved performing multiplex-PCR, specific for particular loci, in which 20 fragments of DNA, around 100 bp (base pairs) long, were amplificated. Each DNA fragment had a polymorphic locus in its sequences.

After performing PCR, the remaining unbound dNTPs were dephosphorylated using Shrimp Alkaline Phosphatase (SAP).

The next stage involved performing iPLEX reaction. On the matrix which was formed during the multiplex-PCR reaction, terminal dideoxynucleotides (ddNTPs), corresponding to the allele variant present in the gene, were bonded to MassEXTEND primers with modified mass and complementary to the sequence, located above the polymorphic locus.

After performing the iPLEX reaction, 15 mg resin was placed on each well of a 96-well plate to bind metal ions, such as Na+, K+, Mg2+, which might cause high background interferences in mass spectra. After centrifugation of the plate (5 min., 4000 rpm), the analyzed material was transferred to SpectroCHIP using Nanodispenser RS1000. Its volume ranged from 3 to 25 nl and bonded with the agent filling each of the 96 wells on the chip.

The first stage of the mass spectrometer analysis included matrix laser desorption, needed for DNA analysis. SpectroCHIP is placed inside the spectrometer, and each place with a DNA sample is ionized with a laser in the vacuum. An energy beam used in laser ionization must be well adjusted so that the particles do not get fragmented but bounced only. Next, the particles proceed to a time-of-flight analyzer. The particles going to the analyzer are accelerated with an electric impulse and start drifting through the analyzer chamber. At the end of the analyzer, an ion detector is connected with a device that registers the time between the acceleration impulse and the moment the particle hits the sensor. A result of the mass-to-charge ratio value corresponds to a value we obtain if we follow a low of physics saying that for an impulse of particular amperage, the time-of-flight of ions gets longer if their molecular weight increases. An analysis of the sample using the MassARRAY mass spectrometer enabled us to obtain raw results presented in the form of mass spectra. The mass spectra, in turn, allowed the identification of polymorphisms in the following studied genes: TGF-β1, TGF-β2, and TGF-β3. The graphs could enable us to determine the reaction yield and probability of the obtained results.

### Statistical analysis

The Kruskal-Wallis rank-sum test was applied to compare asthma control test scores in genotypes, whereas the two-sample Wilcoxon test was used to compare the scores in minor allele carriers (MAC). Due to a large number of comparisons, the *p*-values were adjusted using the Benjamini & Hochberg procedure. Treating the ACT score as continuous, linear regression models were developed for both genotypes and MAC statuses to assess the influence of variables on the ACT score. The backward stepwise selection was based on the Akaike Information Criterion for a fitted parametric model. The overall performance of the models was assessed utilizing multiple R2 and RMSE. At the same time, the influence of certain factors required a calculation of standardized beta coefficients after estimating them using standard deviation. Although measured on a continuous scale, the response variable (ACT score) can be treated as an ordinal variable. Below I check whether the application of ordinal regression (i.e., proportional odds logistic regression) would bring about any changes. The logistic regression was designed in the same manner to test whether the genotypes or MAC statuses will allow predicting reasonable asthma control (ACT≥25). The analysis was performed in STATISTICA 13.1 (Dell, USA) and R programming language (packages: rms, cutopointr, caret).

## Results

**Fig. 1:**
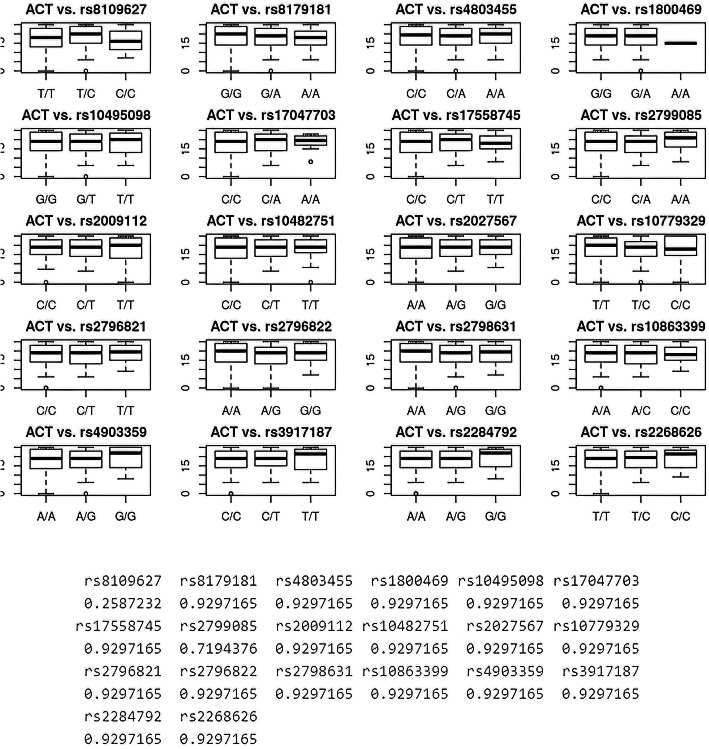
**A** Boxplots showing differences in the median ACT score between genotypes in selected SNPs. A detailed analysis of SNPs of TGF-β1, TGF-β2 and TGF-β3 genes potentially affecting the level of asthma control using the Kruskal-Wallis Test, p with BH correction. p - level of statistical significance. BH - Bonferroni-Hochberg correction. The authors’ own study

Whole genotypes and MACs were tested in TGF-β1, TGF-β2, and TGF-β3 genes. The authors assessed their correlation with the degree of asthma control. It was found that the median of the Asthma Control Test (ACT, score in points) did not differ on genotypes of selected SNPs (*p* > 0.05 for all comparisons, BH [Bonferroni-Hochberg] correction). Details are shown in Fig. 1A.

**Fig. 2:**
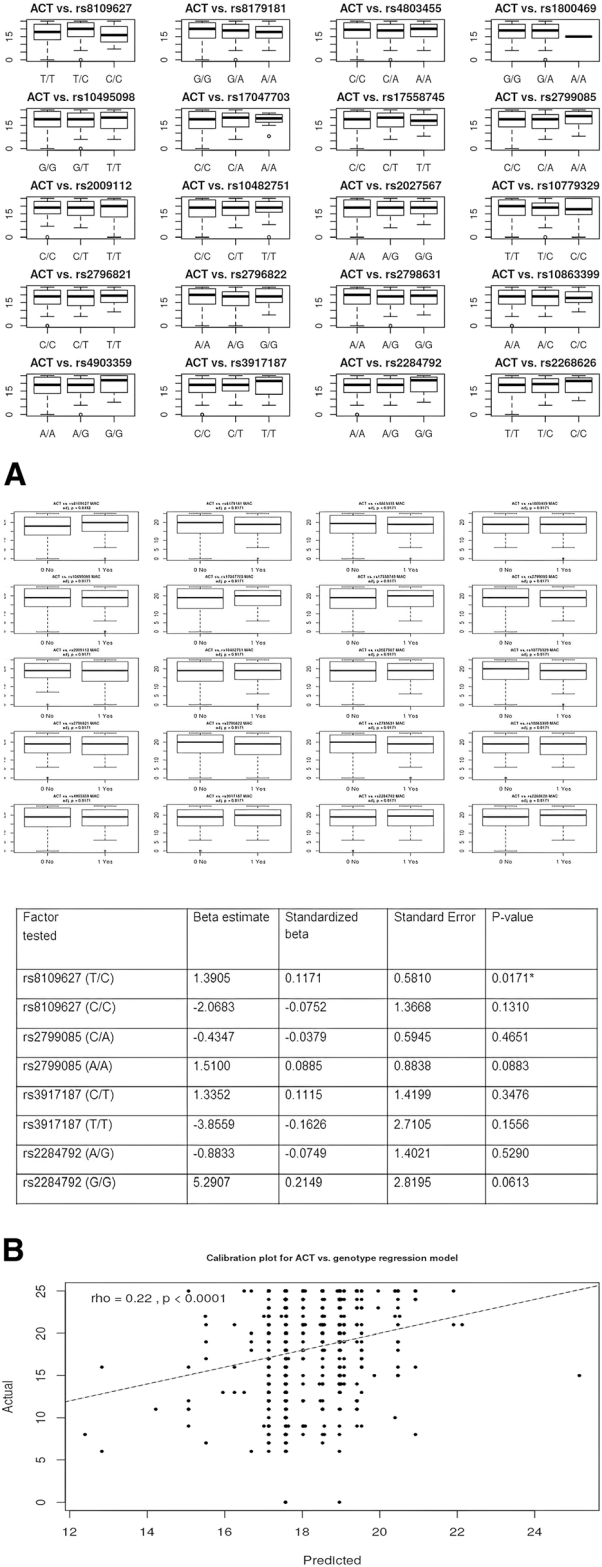
**A** and **B** Coefficients of the linear model for prediction of ACT score. A detailed analysis of MAC in SNPs of TGF-β1, TGF-β2 and TGF-β3 genes potentially affecting the level of asthma control using the U Mann-Whitney Test, with BH correction. p - level of statistical significance. BH - Bonferroni-Hochberg correction. The authors’ own study

After using correction for multiple comparisons, the median ACT did not differ depending on the minority allele carrier (MAC) of selected SNPs (*p* > 0.05 for all comparisons, BH correction). Details are shown in Fig. 2A.

For prediction of ACT based on genotype, the full model without feature selection showed low R2, equal to 0.084 and RMSE equal to 5.47 points. After applying the backward stepwise procedure, rs8109627, rs2799085, rs3917187 and rs2284792 were the only remaining SNPs in the model. Despite the feature selection, the R2 did not improve and was equal to 0.05 with RMSE equal to 5.57 points. The model is shown in Fig. 2A.

The greatest standardized beta coefficient, thus the most significant influence on the predicted ACT, was shown for genotype G/G rs2284792; however, this was not significant in the t-test (*p* = 0.06). T/C genotype in rs8109627 was the only statistically significant variable in the t-test associated with linear modeling. It is further shown in the calibration plot. See Fig. 2B.

Calibration plot showing a relationship between predicted and actual results in the ACT score. Although the correlation is significant, the rho coefficient does not prove a strong relationship

In a similar analysis involving MAC, the final model after feature selection revealed that rs8109627, rs2796822, and rs2798631 are most important. The multiple R2 was, however, low and equal to 0.02, with RMSE equivalent to 5.66 points. The estimates are shown in Table 3.

**Table 3:** Coefficients of the linear model for prediction of the ACT score. The MAC status was considered interesting. Although rs8109627 and rs2796822 show significant association with the ACT score, the effect seems too slight

SNP tested	Beta estimate	Standardized beta	Standard Error	*P*-value
rs8109627	1.1115	0.0957	0.5646	0.0497*
rs2796822	−1.7930	−0.1534	0.8932	0.0454*
rs2798631	1.3661	0.1123	0.9293	0.1423

**Fig. 3:**
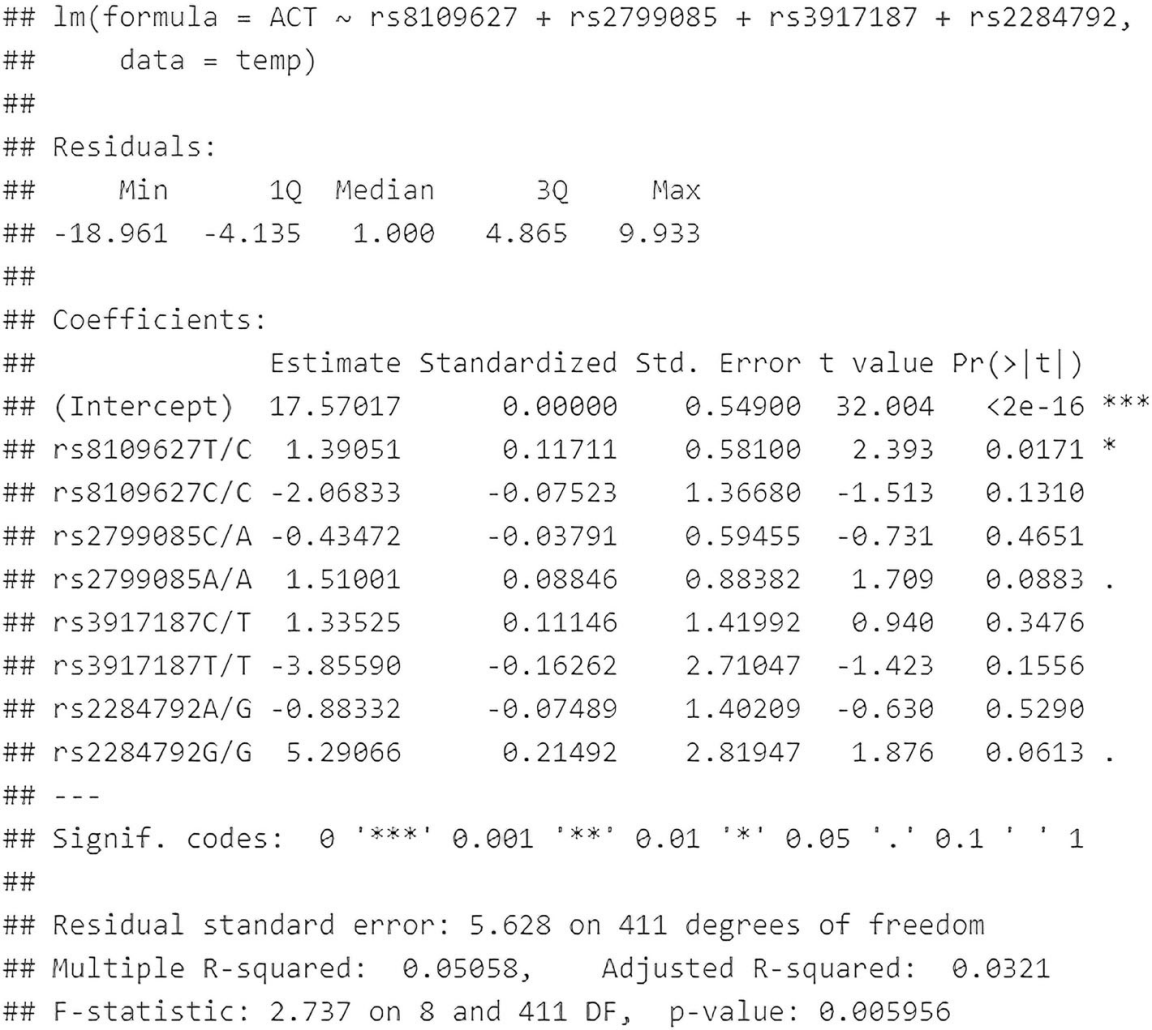
Multivariate linear regression analysis after stepwise backward selection of variables, describing the effect of the studied SNPs genotypes on the level of asthma control (ACT). The authors’ own study

Predictive analysis of the ACT result, depending on the genotypes of the studied SNPs with the application of backward selection, showed that the T / C genotype in rs8109627 is significantly associated with a higher ACT result (standardized beta = 0.11). In general, despite the variable selection, the model was characterized by low efficiency (adjusted R2 0.03). A GVIF analysis also showed that the model could have been described with collinearity of variables. Details are presented in Fig. 3.

**Fig. 4:**
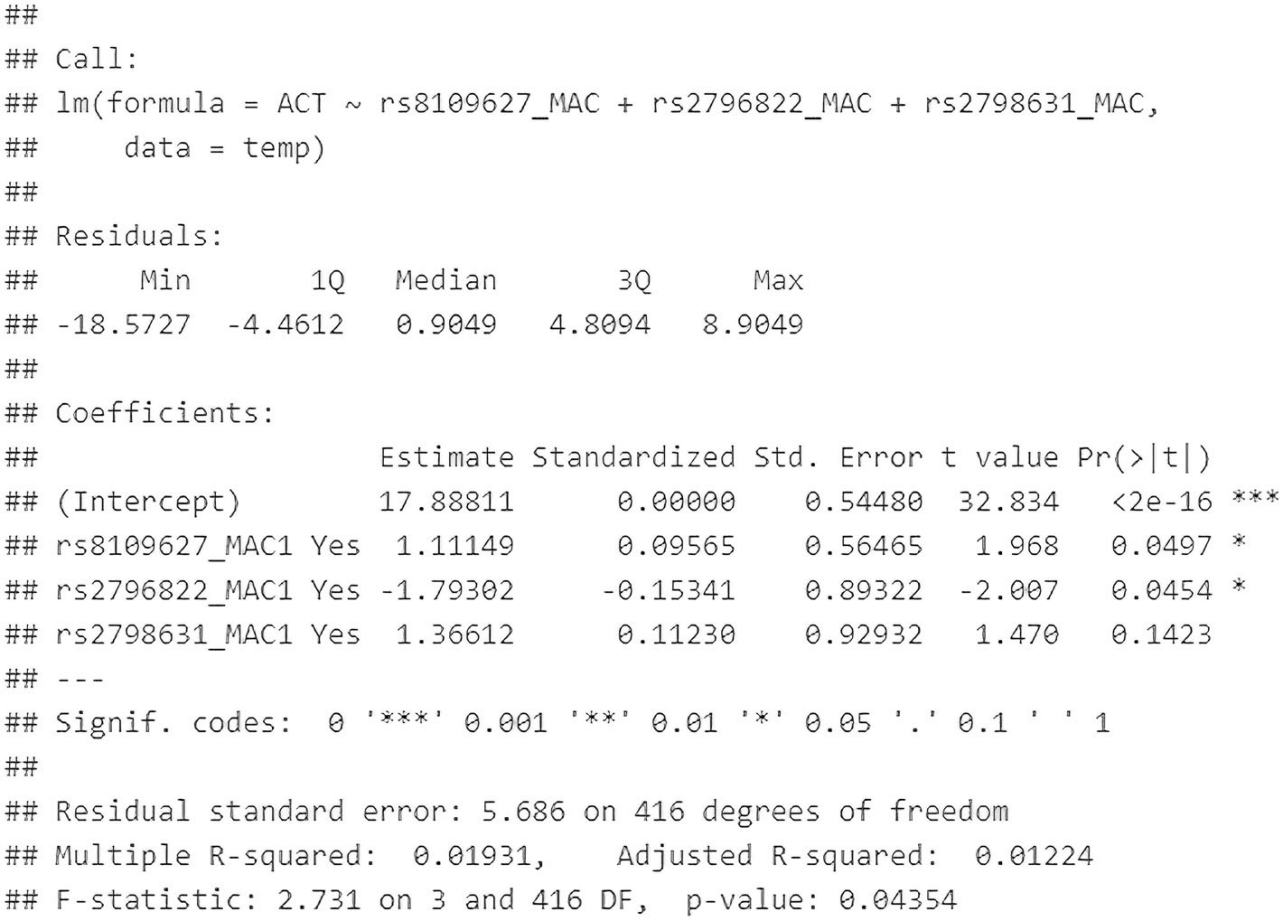
Analysis of ACT result prediction, depending on MAC SNPs (full analysis model including all SNPs). The authors’ own study

Considering only the MAC analysis, in multivariate linear regression analysis, after applying stepwise backward selection of variables, the authors managed to reveal that MAC in rs8109627 is significantly associated with a higher ACT result (better asthma control), and in rs2796822 with a lower ACT result (worse asthma control). The selection of variables showed that MAC is most important in rs8109627, rs2796822, and rs2798631. However, the model was characterized with low efficiency (adjusted R2 0.01). Details of the analysis are presented in Fig. 4.

**Fig. 5:**
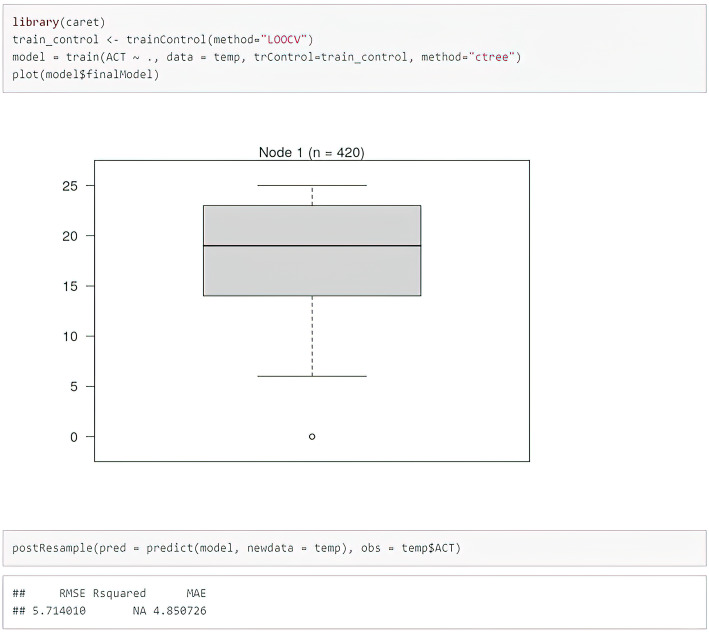
Modeling the c-tree, allowing to prognosticate the degree of asthma control, on the basis of SNPs analysis of TGF-β1, TGF-β2, and TGF-β3 genes. The authors’ own study

The authors made an attempt to create a decision tree (ctree), allowing to prognosticate the degree of disease control in ACT, based on particular SNPs in the studied genes. An attempt to model trees appeared to be unsuccessful in this case. The authors did not manage to obtain results which would be more resistant to over-adjusting after application of other techniques (e.g. ctree - decision trees), as illustrated in Fig. 5.

**Fig. 6:**
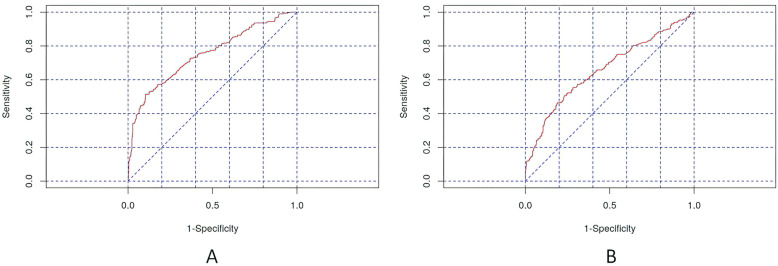
An analysis of prognosis of effective asthma control (ACT> = 25) on the basis of ROC analysis. **A** Logistic regression with the application of all genotypes in SNPs contributed to AUC = 0.749. **B** Logistic regression with the application of all MACs in SNPs contributed to AUC = 0.661. The authors’ own study

An analysis of ROC curves showed that statistical modeling might be helpful in clinical practice. Reasonable asthma control could be predicted by analyzing all twenty SNPs and not by analyzing MAC results in SNPs. These observations are illustrated in Fig. 6.

**Fig. 7:**
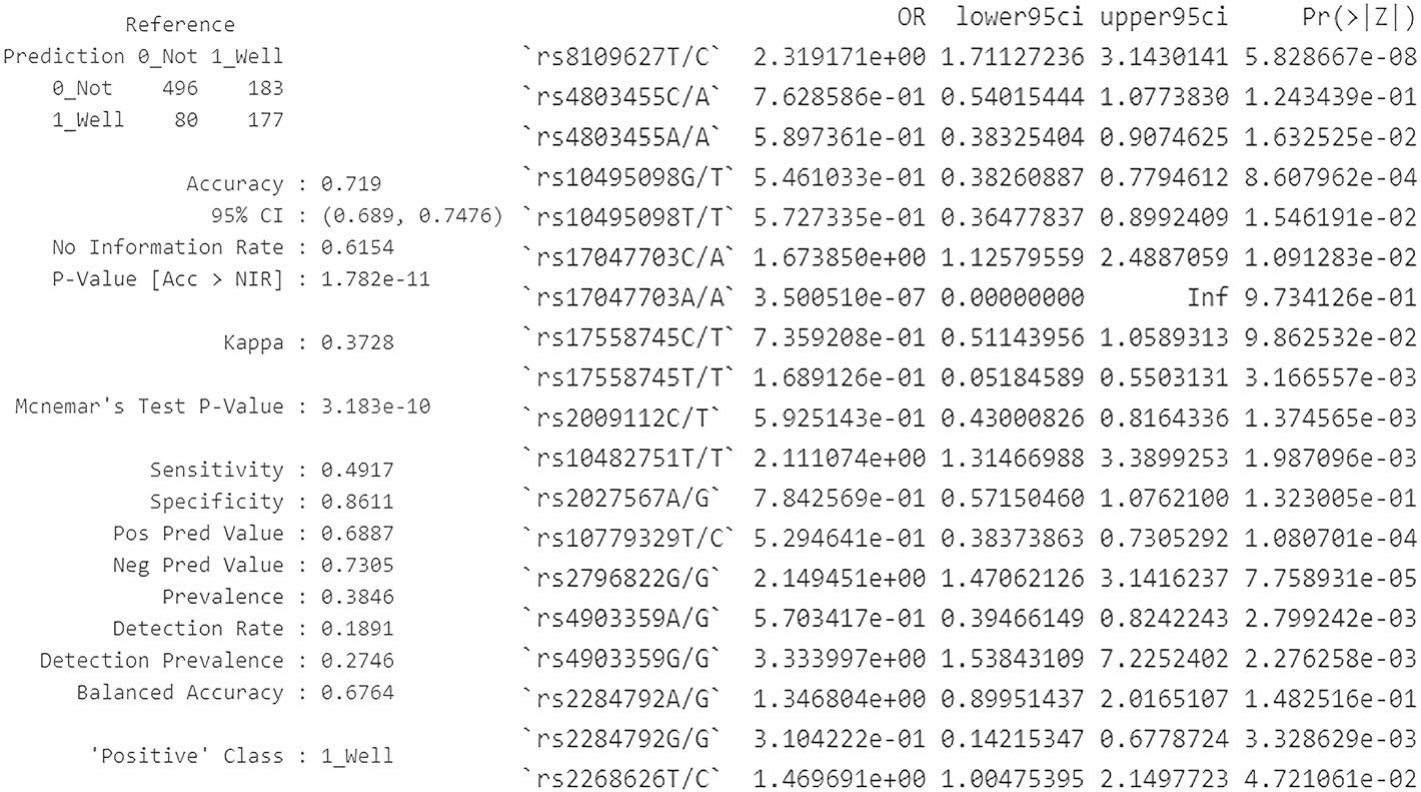
A SMOTE analysis and analysis with the application of backward stepwise selection, enabling to predict good asthma control (ACT> = 25), on the basis of all twenty SNPs. The authors’ own study

Due to the class imbalance, better results were obtained after performing the SMOTE-type modeling. Predicting good asthma control (ACT> = 25) with the use of this method enables to achieve 86.11% specificity and 49.17% sensitivity. Results are shown in Fig. 7.

**Fig. 8:**
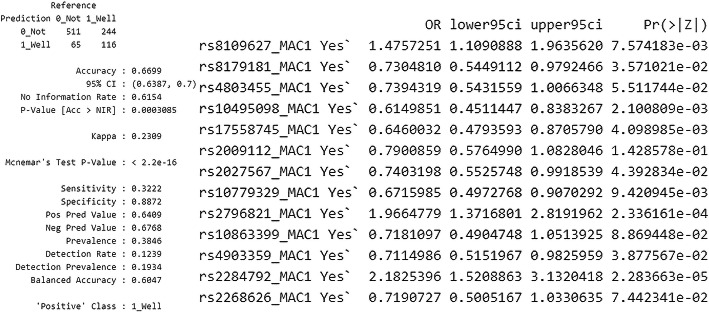
An analysis of the application of a prognostic model on the basis of SNP and MAC genotypes on the entire (training) data set to predict good asthma control. The authors’ own study

Predicting good asthma control is possible with the SMOTE method. It should be noted that both the use of genotypes and MAC allows creating a moderately correct predictive model, which is about 70% efficient on the entire (training) data set. The description is shown in Fig. 8.

## Discussion

The role of polymorphic genes in asthma, especially single ones, has been widely discussed for years in international professional literature [12, 13, 19–21]. Our work, unlike many others, presents a holistic and unique analysis along with a detailed statistical methodology that allows us to assess the effect of twenty SNPs, simultaneously in TGF-β1, TGF-β2 and TGF-β3 genes on the degree of asthma control. Our results are also an attempt to present various statistical approaches to the problem analysis and interpretation of the said problem. The work is unique in this respect [27, 28].

Firstly, we did not note significantly different medians in ACT in genotypes and MACs (adjusted *p*-value > 0.05 for all comparisons). See Fig. 1A. For ACT prediction, based on the genotypes, the full model without feature selection showed low R2 equal to 0.084 and RMSE equal to 5.47 points. After applying the backward stepwise procedure, rs8109627, rs2799085, rs3917187, and rs2284792 were the only remaining SNPs in the model. Despite feature selection, the R2 did not improve and was equal to 0.05, with RMSE equal to 5.57 points. See Fig. 2A. The greatest standardized beta coefficient, thus the greatest influence on the predicted ACT, was shown for genotype G/G rs2284792; however, this was not significant in the t-test (*p* = 0.06). See Fig. 2A. The only statistically significant variable in the t-test and associated with linear modeling was T/C genotype in rs8109627. It is further shown in the calibration plot. See Fig. 2B. In a similar analysis involving MAC, the final model after feature selection revealed that rs8109627, rs2796822, and rs2798631 are most important. The multiple R2 was, however, low and equal to 0.02, with RMSE equal to 5.66 points. The estimates are shown in Table 3.

Next, in order to check whether the genotypes or MAC statuses will allow predicting reasonable asthma control (ACT≥25), the logistic regression was designed in the same manner. The analysis allowed us to conclude that after performing the feature selection, the final model included only rs8109627 and rs17047703 [27, 28]. See Supplementary File No 1.

It is difficult to discuss the issues described above because currently available databases (e.g., https://www.ncbi.nlm.nih.gov/pubmed/?term=SNP+TGF+asthma+control) do not have analyses of polymorphic forms of TGF-β1, TGF-β2, and TGF-β3 genes, which influence the degree of asthma control. It also shows the unique nature of our work. Although literature reports an impact of single SNPs on the clinical course of asthma, there are no studies on the association between the TGF-β gene and the control of asthma symptoms [12, 13, 19, 20].

Secondly, we examined the possibilities of assessing correlations with polymorphic forms of TGF-β genes using various statistical methods. We showed that in the multivariate linear regression analysis model, after using backward stepwise selection of variables, the occurrence of the T/C genotype in rs8109627 is significantly associated with a higher ACT score; however, this effect is not spectacular (beta standardized = 0.11). It should be pointed out here that it has not been well documented that this rs8109627 in the TGFβ1 gene plays a role in increasing the risk of asthma. We obtained slightly different results after analyzing not SNP but MAC in TGF-β genes. After backward stepwise selection of variables, the multivariate linear regression analysis of MAC showed that MAC in rs8109627 is significantly associated with a higher ACT™ result, and in rs2796822 - with a lower one. Interestingly, this rs2796822 in the TGF-β2 gene occurs with different frequencies [1.71 (1.07,2.71) *p* = 0.022] in patients with non-severe and severe asthma. The risk of severe asthma in MAC carriers for the SNP mentioned above was 71% higher (the author’s data in the publication procedure). A further selection of variables confirmed that MAC is most important in rs8109627, rs2796822, and rs2798631, but this model was characterized with low efficiency (adjusted R2 = 0.01). SNPs rs8109627 and rs2798631 correlated with a clinically significant higher level of asthma control (controlled asthma) in the ACT™ Test, whereas rs2796822 was associated with a lower result (loss of disease control). See Fig. 2B. Nevertheless, this is the first report about the role of these three tagging SNPs in asthma in TGF-β1, TGF-β2, and TGF-β3 genes. An attempt to optimize the prediction of good asthma control was also made (ACT> = 25). Due to the class imbalance, better results were obtained after performing the SMOTE-type modeling than after applying multivariate linear regression analysis. For details, see Figs. 6, 7, and 8. Conducting analysis using various statistical models enables both genotypes and MAC, which creates a moderately correct prognostic model that is about 70% efficient on the entire (training) data set (Fig. 8).

In Supplementary File No 1, we checked whether the genotypes or MAC statuses would allow predicting good asthma control (ACT≥25); the logistic regression was designed in the same manner. The conducted procedure concluded that after feature selection, the final model included only rs8109627 and rs17047703. See Supplementary File No 1. The role of tagging SNP rs8109627 in the TGF-β1 gene and rs17047703 in TGF-β2 had not yet been described. We were the first in the above analysis to test it with advanced statistical methods [27, 28].

In our work, we analyzed twenty tagging SNPs in TGF-β1, TGF-β2, and TGF-β3 genes. We critically interpreted the results with statistically different methods. It should be noted that only three SNPs, such as rs8109627, rs2796822, and rs2284792, play a significant clinical role in the regulation of asthma control. This is an important discovery since the role of these SNPs in the TGF-β gene in asthma had not been previously described. It should be critically stated that the prognostic efficiency of different statistical models was up to 70%. Nevertheless, the work, not carried out before, is a unique and holistic analysis of twenty polymorphic forms in TGF-β1, TGF-β2, and TGF-β3 genes using state-of-the-art techniques of molecular biology (MassARRAY® system).

## Limitations

Both the use of genotypes and MAC allow the creation of only a moderately efficient prognostic model. In the multivariate linear regression analysis, we managed to show that only single genotypes significantly correlated with higher ACT results after using backward stepwise selection of variables. However, this effect was not spectacular. In general, despite the variable selection, the efficiency of the model was still low. A GVIF analysis also showed that the model could have been characterized with collinearity of variables.

Moreover, the selection of variables showed that MAC was most important in three SNPs, but this model was not also highly efficient. The authors did not manage to obtain results that would be more resistant to over-adjusting with the application of other techniques (e.g. ctree - decision trees). Due to the class imbalance, better results were obtained after performing the SMOTE-type modeling. After completing the SMOTE-type modeling, the logistic regression model was trained on the whole dataset. The application of SMOTE vastly inflates those results. By creating new cases that are similar to the original ones, the performance was only minimally inflated. According to the authors of the above statistical analyses, linear regression models are the only ones that can be used in this type of scientific study. The small size of the surveyed populations is another limitation of the study. It would be advisable to verify our theses on subpopulations consisting of about 1000 respondents. Our results should be treated as preliminary.

## Conclusion

This study is the first one ever conducted in the Polish population. It attempts to analyze the functional impact of twenty SNPs of TGF-β1, TGF-β2, and TGF-β3 genes on the degree of asthma control, assessed using the international ACT™ Questionnaire. We showed new relationships between the occurrence of SNP rs8109627 in the TGF-β1 gene (improved disease control - controlled asthma) and rs2796822 in the TGF-β2 gene (worsened disease control - uncontrolled asthma) and a significantly changed level of asthma control. This is a particularly important observation because the TGF-β gene affects eosinophil levels, bronchial hyperreactivity and obstruction, and clinical signs of asthma. In contrast, the TGF-β1–3 gene complex is an essential regulator of the immune response in asthma. We also proposed a new statistical analysis model (multivariate linear regression analysis with backward stepwise selection of variables and the SMOTE model) to assess the effect of many SNPs, simultaneously in three TGF-β genes, on disease control. The analyzed results and methods were a subject of our internal discussion, during which we identified those that may be clinically useful. The presented results constitute an innovative approach to understanding the importance of SNP forms in TGF-β1, TGF-β2, and TGF-β3 genes in the regulation of asthma control.

## Supplementary Information

**Additional file 1.**

**Additional file 2.**

## Data Availability

All data and materials are presented in the section of the article. The authors will always provide other indicated data on request. Raw data were attached as the supplementary dataset.
