# Supplementary material for: TGF-β gene polimorphisms as risk factors for asthma control among clinic patients
Source: J Inflamm (Lond). 2021 Oct 7;18:28. doi: 10.1186/s12950-021-00294-4 (PMC8499525; doi:10.1186/s12950-021-00294-4)
Supplement: Supplementary file 1 — Additional file 1. [file 12950_2021_294_MOESM1_ESM.doc]

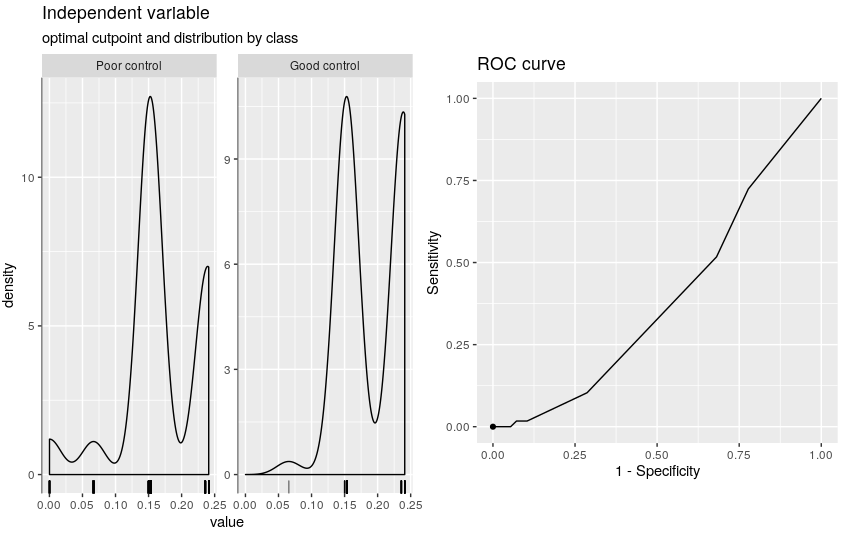


This model gives very poor performance. After tinkering with multiple set of features, multiple modelling methods – It was not able to find any association of SNPs with good or poor asthma control.
